# Sexual competition and kin recognition co-shape the traits of neighboring dioecious *Diospyros morrisiana* seedlings

**DOI:** 10.1038/s41438-021-00598-9

**Published:** 2021-07-01

**Authors:** Yulin He, Han Xu, Hanlun Liu, Meiling Luo, Chengjin Chu, Suqin Fang

**Affiliations:** 1 State Key Laboratory of Biocontrol, School of Life Sciences, Sun Yat-sen University, Guangzhou, 510275 China; 2 Research Institute of Tropical Forestry, Chinese Academy of Forestry, Guangzhou, 510520 China

**Keywords:** Evolutionary ecology, Molecular ecology, Plant ecology

## Abstract

Plants respond differently to the identity of their neighbors, such as their sex and kinship, showing plasticity in their traits. However, how the functional traits of dioecious trees are shaped by the recognition of neighbors with different sex and kinship remains unknown. In this study, we set up an experiment with different kin/nonkin and inter/intrasexual combinations for a dioecious tree species, *Diospyros morrisiana*. The results showed that plants grew better with nonkin and intrasexual neighbors than with kin and intersexual neighbors. Kin combinations had significantly shorter root length in the resource-overlapping zone than nonkin combinations, suggesting that kin tended to reduce competition by adjusting their root distribution, especially among female siblings. Our study suggested that the seedling growth of *D. morrisiana* was affected by both the relatedness and sexual identity of neighboring plants. Further analysis by gas chromatography-mass spectrometry showed that the root exudate composition of female seedlings differed from that of male seedlings. Root exudates may play important roles in sex competition in dioecious plants. This study indicates that sex-specific competition and kin recognition interact and co-shape the traits of *D. morrisiana* seedlings, while intrasexual and nonkin neighbors facilitate the growth of seedlings. Our study implies that kin- and sex-related interactions depend on different mechanisms, kin selection, and niche partitioning, respectively. These results are critical for understanding how species coexist and how traits are shaped in nature.

## Introduction

How plant species coexist with their neighbors determines the community structure, and understanding these interactions has attracted much research attention^1,2^. Having limited dispersal ability and a sessile lifestyle, plants often coexist with few neighbors^3,4^, with whom they engage in both above- and belowground interactions. For example, bean and pea species outcompete neighbors for light by elongating their aboveground stems^5^, and maize roots overproliferate around nutrient-rich patches to outcompete neighbors^6^. Therefore, plant species are known to mediate this competitive response in the presence of neighbors with different identities by adjusting their fundamental morphology^7^, physiological traits^8^, life history traits^9^, or resource allocation strategies^10^. However, this capacity for neighbor identity recognition is still poorly understood, particularly with regard to neglecting individual importance in the community.

Although neighbor-identity recognition is known among dioecious species, it is often ignored^2,11,12^. Dioecious species have separate male and female individuals, and this distinction plays a significant role in maintaining stability in the structure and function of terrestrial ecosystems^13^. Unfortunately, the differences in competitive abilities between the sexes remain poorly understood. For example, some studies have demonstrated that females are competitively superior to males in growth and/or survival at high densities^14,15^, while others have shown that males possess superior resistance and adaptive abilities under environmental stress^16^. If dioecious plants have the capacity to recognize the sex of conspecific neighbors, they might modulate the intensity of their intersexual and intrasexual competition.

Among dioecious plants, potential competition between the sexes complicates kinship interactions. Mercer and Eppley^11^ demonstrated that kin and sexual interaction significantly co-affect different traits of *Distichlis spicata*. Competitive traits such as the root distribution^7,17–19^ and leaf distribution^20,21^ have also been used to identify kin recognition. However, few studies have considered the role of sex and the relatedness of neighbors in the process of dioecious species competition, and little is known about their interaction^11,12^.

**Fig. 1:**
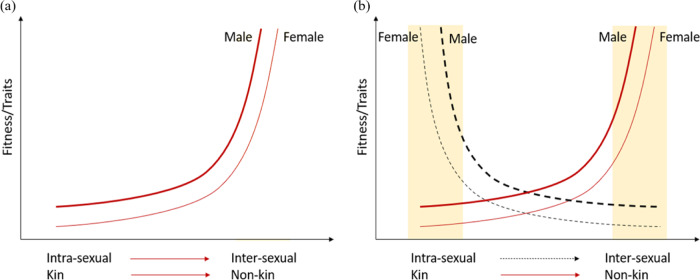
Theoretical hypothesis for the fitness of females and males in paired plantings, with variation in kinship and for intra- and intersexual combinations. **a** Where intersexual combinations have higher fitness/traits than intrasexual combinations and nonkin have higher fitness/traits than kins, and **b** where intrasexual combinations have higher fitness/traits than intersexual combinations and nonkin have higher fitness/traits than kins. The yellow bars indicate the data ranges of intrasexual and kin combinations or that intersexual and nonkin combinations have larger fitness/traits than the others

Spatial segregation of the sexes (SSS) is fairly common in dioecious plants, occurring in over 30 species from multiple families^22^. One of the mechanisms proposed to explain SSS in angiosperms is the niche partitioning hypothesis, which suggests that plants have evolved to occupy different niches because intersexual competition is greater than intrasexual competition^4,17,23,24^. In addition, the kin selection theory suggests that plants save resources by cooperating with kin and competing with nonkin, leading to fewer competitive traits, higher fitness, and contributing to the evolution of kin selection^25^. The kin selection theory and niche partitioning hypothesis together suggest a hypothetical framework of our study that nonkin/intersexual competition is greater than kin/intrasexual competition (Fig. 1a) and that plants will allocate more resources into traits conferring competitive advantages with increasing competition intensity.

The various organic and inorganic substances released by plant roots into the rhizosphere environment through metabolic and nonmetabolic pathways are root exudates^26^. Root exudates not only have nonspecific allelopathic effects but also have specific effects in mediating the perception of the identity of neighbors^18,27^. For example, root exudates of *Arabidopsis thaliana* significantly increased the lateral root number of distantly related plants but had no effect on close relatives^27^. In addition, root exudates are also involved in the growth and interaction of dioecious plants *D. spicata*^11^ and mulberry (*Morus alba*) seedlings^28^. Adding the root exudates of intersexual plants to a culture solution of *D. spicata* can reduce plant biomass and increase the root-to-shoot ratio^29^. Female mulberry trees increased their root system size when exposed to intersexual root exudates^28^. However, it remains largely unknown what specific compounds in root exudates are responsible for neighbor recognition in dioecious species.

We tested this hypothesis by setting up kin/nonkin and intersexual/intrasexual treatments together for a dioecious tree species, *Diospyros morrisiana*. Plants were grown in a homogeneous sterile gel medium system as a novel way to address kinship and sex interactions without the influence of soil microorganisms. Our aims were to address the following questions: (1) How do dioecious species respond to different sexual neighbors? (2) How do dioecious species respond to kin or nonkin neighbors? (3) Do plants compete more in nonkin/intersexual combinations in a broad sense than in kin/intrasexual combinations? (4) What are the mechanisms of kin and sex recognition?

## Results

### Growth difference between male and female *Diospyros morrisiana* seedlings in monoculture

**Fig. 2:**
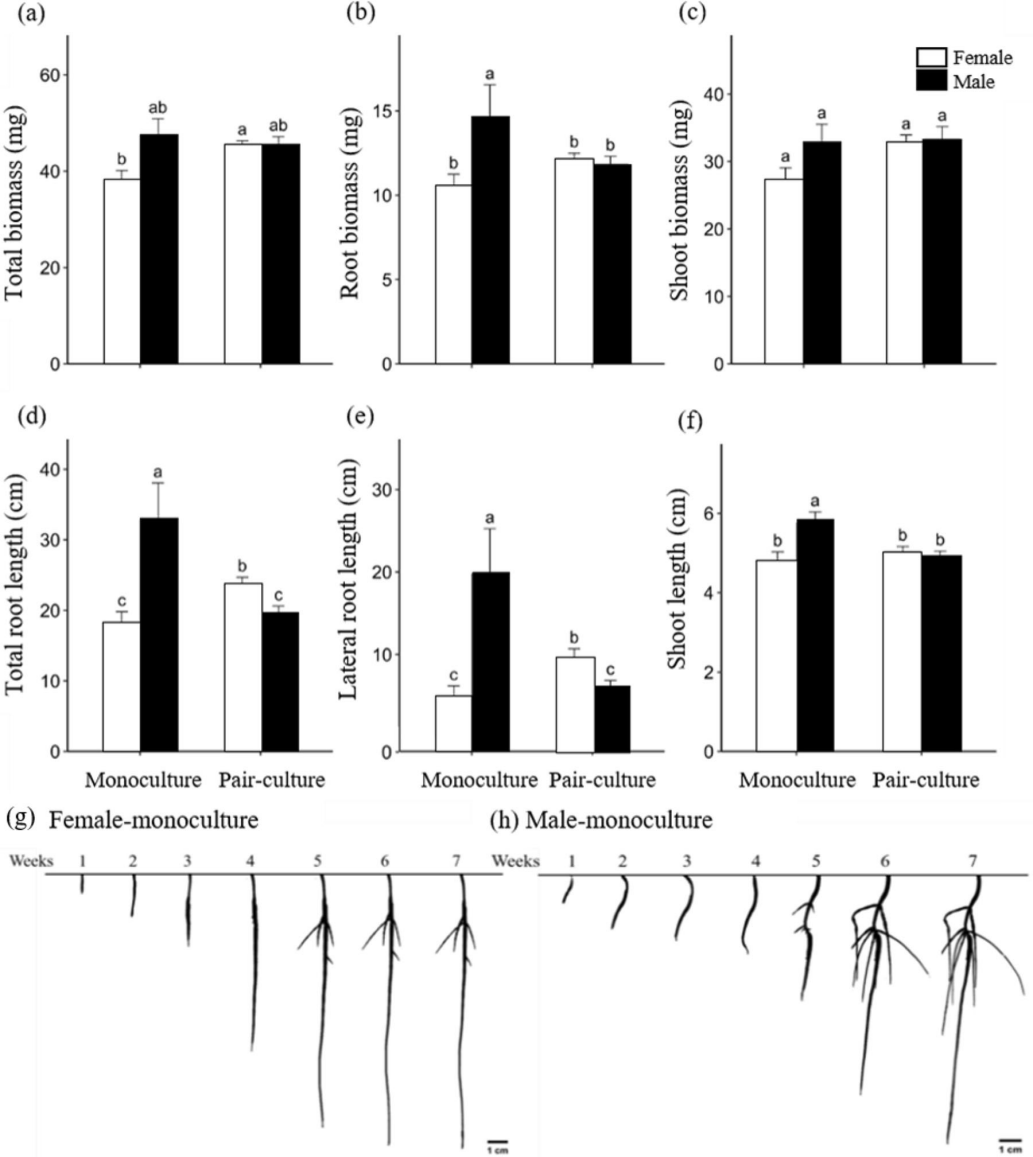
Biomass and growth characteristics of female and male individuals in monoculture and pair-culture treatments 7 weeks after germination. **a** Total biomass, **b** root biomass, **c** shoot biomass, **d** total root length, **e** lateral root length, and **f** shoot height. Treatments with the same letter do not differ significantly (*p* < 0.05). The bottom row shows a generalized representative two-dimensional root system under monoculture for **g** female and **h** male *D. morrisiana* plants 7 weeks after germination

In single plantings, *D. morrisiana* male seedlings had larger root systems than female seedlings (Fig. 2, Table S1). When grown alone, at the 7th week, males had significantly larger root biomass (*P*-value = 0.0185; Fig. 2a, b) and total biomass (*P*-value = 0.0338; Fig. 2a-a) than females but did not significantly differ from single females in their shoot biomass (*P*-value = 0.109; Fig. 2a–c). The total and lateral root length and shoot height of single males were also significantly larger than those of single females (Fig. 2d–h, Table S2).

### Seedling growth difference between monoculture and paired culture

Compared with single individuals, male seedlings grown with neighbors had lower root biomass (Fig. 2b), total root length (Fig. 2d), lateral root length (Fig. 2e), and shoot height (Fig. 2f), while females grown with neighbors had larger total biomass (Fig. 2a), total root length (Fig. 2d), and lateral root length (Fig. 2e).

**Fig. 3:**
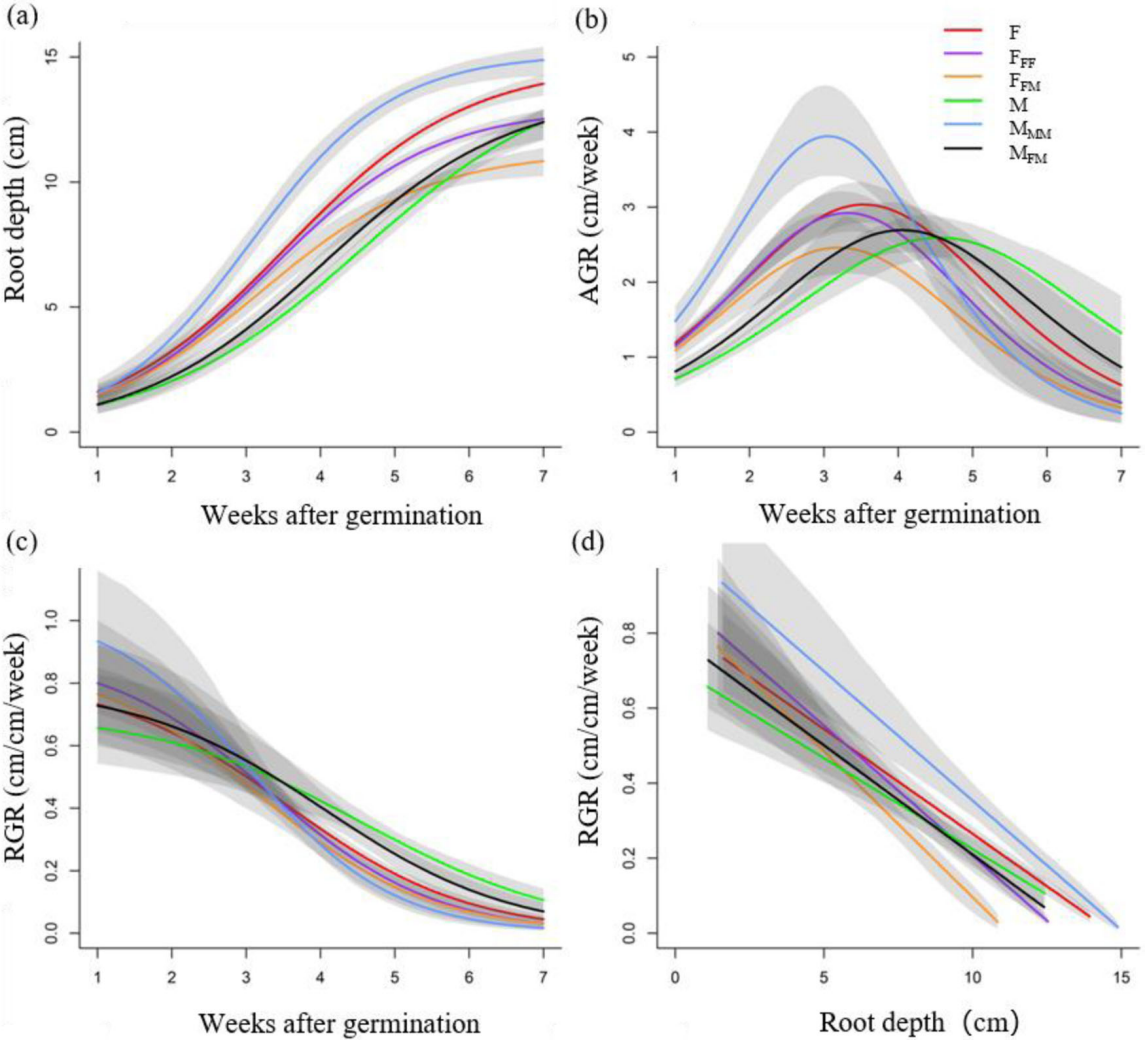
Predicted responses of *D. morrisiana* from the logistic model grown in monoculture and in combination with different sexes. **a** Root depth as a function of time, **b** absolute root depth growth rate (AGR) as a function of time, **c** relative root depth growth rate (RGR) as a function of time, and **d** relative growth rate (RGR) as a function of root depth. F represents a single female, F_FF_ represents an intrasexual (female–female) planting combination (FF), F_FM_ represents an intersexual (female–male) interaction (FM), M represents a single male, M_MM_ represents a male in the intrasexual interactions (MM), and M_FM_ represents a male in the intersexual interactions (FM). Gray shading indicates 95% confidence intervals for the accumulated root growth and growth rates, as derived from population prediction intervals

During the entire 7 weeks, both females and males grown in paired combinations had greater modeled root depth in intrasexual (F-F and M-M) than intersexual combinations (F-M) (Fig. 3a). The absolute growth rate (AGR) was a unimodal function of time, reaching a maximum at approximately weeks 3–5, while the relative growth rate (RGR) had a decreasing sigmoidal response in all combinations (Fig. 3b, c). Males grown with other males (M_MM_) had larger AGRs than those grown with other females (M_FM_) in the first 4 weeks (Fig. 3b), and M_MM_ had larger RGRs than M_FM_ in the first 3 weeks (Fig. 3c). F_FF_ had larger AGRs than F_FM_ (Fig. 3b), and the RGRs of F_FF_ were slightly higher than those of F_FM_ (Fig. 3c) over the entire 7 weeks.

In all combinations, the RGRs declined linearly with increasing root depth (Fig. 3d). The RGRs of M_MM_ and F_FF_ were always larger than those of M_FM_ and F_FM_, respectively. However, the root width growth dynamics did not differ among any planting combinations (Fig. S2). Similarly, no significant differences were observed in biomass or in the other tested shoot and root traits among all the planting combinations (Fig. S3).

### Kin interactions in females and males

**Fig. 4:**
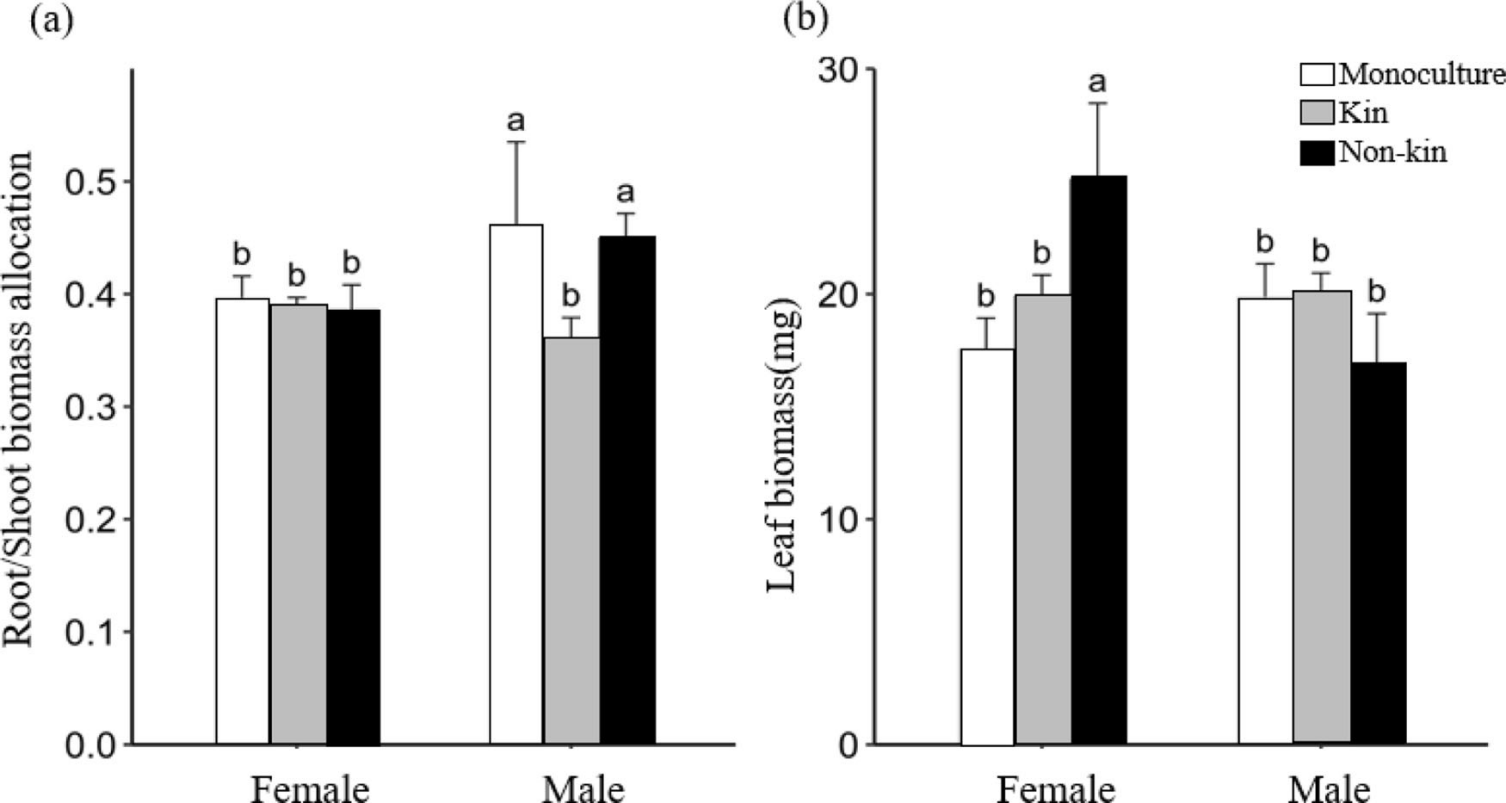
Biomass allocation of *D. morrisiana* when grown with different kin. **a** Root/shoot biomass and **b** leaf biomass. Mean + 1 SE are given without data transformation. Different letters indicate statistically significant differences (*p* < 0.05) among different treatments

**Fig. 5:**
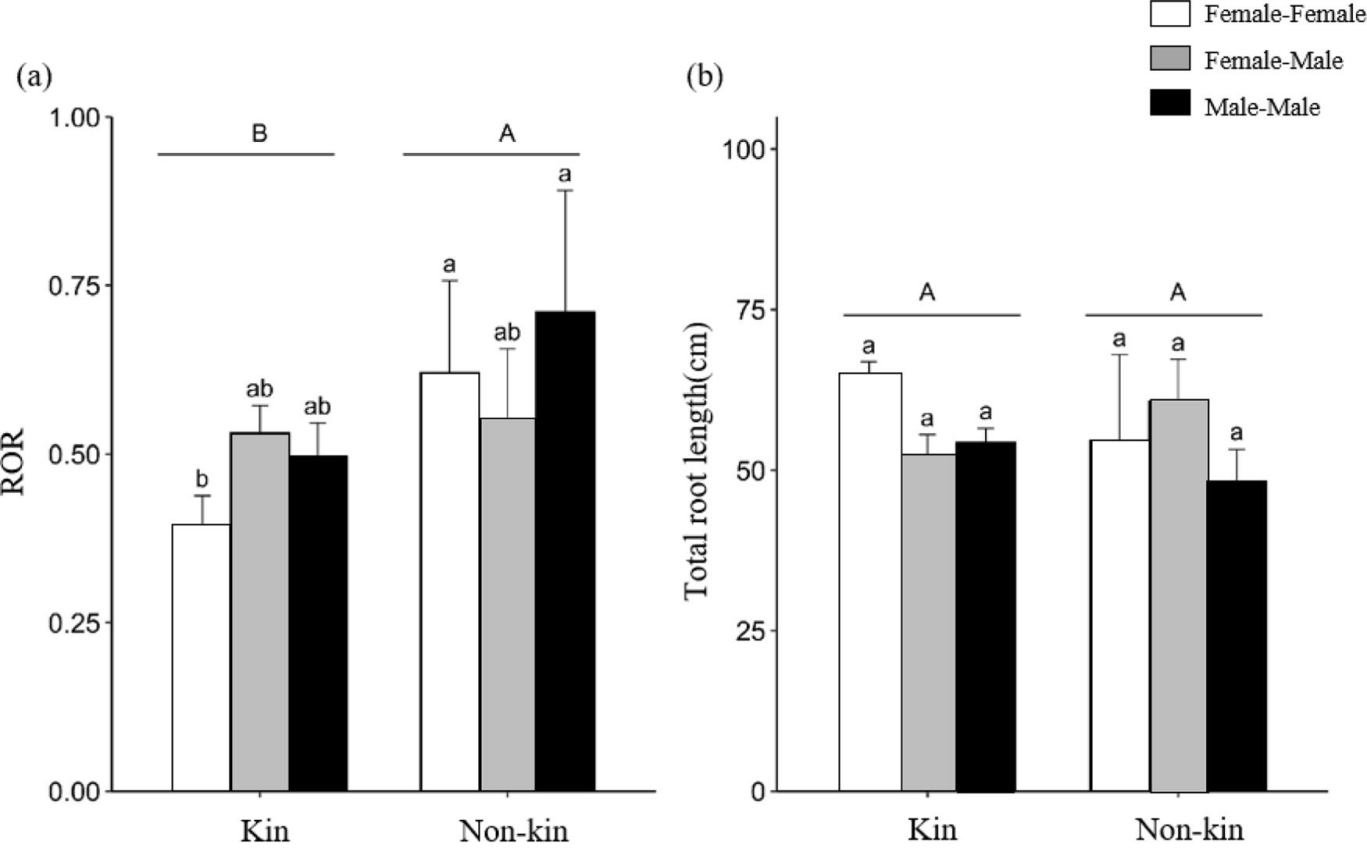
Root growth under different sex and kin combinations. **a** Ratio of overlapping total root length (ROR) in the resource utilization overlapping zone and **b** total root length. Mean +1 SE are given without data transformation (*N* = 65). Different letters indicate significant differences (*p* < 0.05) between treatments: upper-case = kin vs. nonkin; lower-case = sex combinations

Male seedlings paired with nonkin had significantly larger root/shoot biomass than those grown with kin (Fig. 4a). Female seedlings interacting with nonkin had significantly higher leaf biomass than those grown with kin (Fig. 4b). Moreover, paired nonkin plantings had higher root distribution ratios in the resource overlapping zone (ROR) than those planted with kin, especially in the F-F treatments (Fig. 5a). There was no significant difference in the total root length among the six treatments, kinship treatments or sex treatments (Fig. 5b).

### Interactions between sex and kinship

**Fig. 6:**
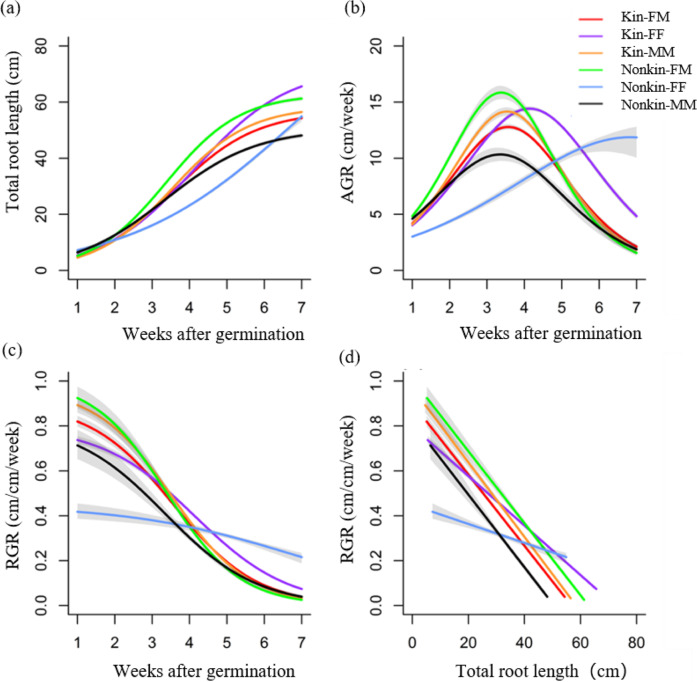
Predicted total root length values of *D. morrisiana* from the logistic model when interacting with different sexes and kin. **a** Total root length, **b** absolute growth rate (AGR), **c** relative growth rate (RGR) on a time basis, **d** relative growth rate (RGR) on a total root length basis. Gray shading indicates 95% confidence limits in the estimate for the accumulated growth and the two growth rates, as derived from population prediction intervals

The total root length in nonkin-FM was significantly larger than that in kin-FM after 2 weeks (Fig. 6a) because the AGRs of nonkin-FM were larger than those of kin-FM in the first 5 weeks (Fig. 6b) and the RGRs of nonkin-FM were larger than those of kin-FM in the first 3 weeks (Fig. 6c). Along with the increase in total root length, the RGRs of nonkin-FM were higher than those of kin-FM over the whole 7 weeks (Fig. 6d).

In the three kin combinations, there were no significant differences in total root length until the 5th week (Fig. 6a). After five weeks, differences were observed in the order of kin-FF > kin-MM > kin-FM (Fig. 6a). The absolute growth rate (AGR) and relative growth rate (RGR) of the three kin combinations started to bifurcate during the 3rd and 4th weeks. The AGR of kin-FF was larger than those of the other two combinations after the 4th week (Fig. 6b, c).

In the three nonkin combinations, nonkin-FM had the largest total root length from the 3rd week (Fig. 6a), which can be directly explained by the largest AGR and RGR recorded for nonkin-FM in the first 4 weeks (Fig. 6b, c). The total root length of nonkin-FF exceeded that of nonkin-MM after week 6 (Fig. 6a), since their AGRs and RGRs differed after 4 weeks (Fig. 6b, c).

### Gas chromatography-mass spectrometry (GC-MS) analysis of root exudates

The GC-MS results showed that the root exudate components were different among the 12 samples. We analyzed the compounds that were detected in at least two samples of each sex and obtained 34 compounds that were identical between female and male root exudates (Table S3). The peak area percentage of “silane diol, dimethyl-” was the highest, accounting for 26.39% and 21.81% of female and male root exudates, respectively. Eight unique compounds in female root exudates and four unique compounds in male root exudates were also found (Table S3).

When we analyzed the compounds that were detected in at least three samples of each sex, 14 compounds were identical. The peak area percentages of decanal and nonanal in the male root exudates were significantly higher than those in the female root exudates (Fig. S4). Other compound peak area percentages were not significantly different between female and male root exudates.

## Discussion

### Kin- and sex-related interactions depend on different mechanisms

Our study suggested that seedling growth of *D. morrisiana* was affected by both relatedness and sexual identity in neighboring plants. Kin recognition and sexual competition also led to contrasting fitness consequences in *D. morrisiana* seedlings (Fig. 1b). *D. morrisiana* seedlings grew better with nonkin and intrasexual neighbors than with kin and intersexual neighbors. This supports our null hypothesis for kin interactions but not for competition between the sexes.

Kin selection was evident in *D. morrisiana* seedlings, which matched our hypothesis (Fig. 1b). When paired with nonkin neighbors, females and males had significantly higher leaf biomass and root/shoot biomass ratios, respectively, than those paired with kin neighbors. This not only supports kin recognition in *D. morrisiana* seedlings but also suggests stronger competition between nonkin than kin individuals. Male seedlings competing with nonkin individuals had significantly higher biomass allocation (root/shoot biomass) than those grown with kin. Female seedlings competing with nonkin individuals had larger leaf biomass than when grown with siblings, which may allow females to increase light reception.

Kin combinations also had significantly shorter root length in the resource-overlapping zone than nonkin combinations, suggesting that kin tend to reduce competition by adjusting their root distribution, especially between female siblings. These results are contrary to those of Fang et al.^7^, who found that rice plants interacting with neighbors of the same genotype had significantly higher root overlap length than those of a different genotype. The reduced overlap in roots in the resource zone might be due to differences in life history traits between rice and *D. morrisiana* used in our study.

On the other hand, sexual recognition could not be explained by the niche partitioning hypothesis (Fig. 1b), which is not consistent with the hypothesis shown in Fig. 1a. We found that both females and males had greater root depth in intrasexual combinations than in intersexual combinations, which may be caused by males and females demanding different resources as a consequence of different demands for reproduction. The same sexes may require more of the same resources, indicating that intrasexual competition is greater than intersexual competition in *D. morrisiana* seedlings. The seedlings used in this study were only in the vegetative state, and there may be some innate differences between the sexes. The dioecious grass *Distichlis spicata* exhibits a similar trend, producing substantially less biomass when grown with intersexual than intrasexual competitors^24^. However, the biomass and root/shoot biomass ratio in *D. morrisiana* seedlings did not differ between intersexual and intrasexual combinations over the culture period. This suggests an interesting possibility in which plants may adapt to their environment by adjusting their root architecture instead of changing their root biomass allocation in the seedling stage.

Moreover, trait variations between female and male individuals in response to the presence of neighboring plants is considered to be an important factor driving sex-specific growth patterns^15^. Females increase their lateral root length when competing with neighbors, indicating an asymmetric increase in competitive ability between females and males in sexual competition. The formation of lateral roots presumably improves sink strength, allowing female plants to increase nutrient intake and water acquisition. Such asymmetric sex-specific competition has been found in *Osyris quadripartite*, in which males were found to be more inhibited when competing with neighboring females^30^. Similarly, *Distichlis spicata* seedlings, regardless of sex, were six times larger when grown with male than female intraspecies plantings, suggesting that females had stronger competitive effects on neighbors than males^15^.

### Root exudates involved in the process of sex competition in dioecious plants

Among the 8 female-specific compounds, “1,2-benzenedicarboxylic acid, bis(2-methylpropyl) ester (DIBP)”, and “pyridine” are 2 substances that are clearly known to have allelopathic effects^31,32^. This indicates that the root exudates of female seedlings have strong allelopathy.

Nonanals and decanals play an important role in resisting pathogens^33,34^. High concentrations of nonanal have inhibitory effects on Penicillium and Botrytis cinerea, which can reduce the rot rate of tomato plants^33^. Decanal significantly inhibited the growth of Pseudomonas swelling at a concentration of 0.12 g/L. When its concentration is 0.24 g/L, it has a good control effect on blue mold on apples and pears and can inhibit the production of aspergillin^34^. The peak area percentages of decanal and nonanal in the male root exudates were significantly higher than those of female root exudates, which may imply that male seedlings are more resistant to pathogens than female seedlings.

The differences in the composition and content of root exudates between females and males may cause sex differences in allelopathic effects and pathogenic resistance, which may indirectly affect the response of dioecious plants to neighbors of different sexes. Thus, root exudates are likely to participate in the process of sex competition in dioecious plants.

We conclude that kin selection and niche partitioning mechanisms are together responsible for the sexual competition and kin recognition of *D. morrisiana* seedlings. Future work should look to understand the fitness consequences of kin recognition and sex competition among dioecious species of different ecotypes, which could play important roles in the evolution of coexistence and reproductive strategies in dioecious plants. In addition, it is important to highlight that the mechanism identified may differ from natural conditions because of the absence of microbial communities in the gel cultivation system used in this study. Sex-specific mycorrhizal colonization has been found in several dioecious and gynodioecious plants^35–37^. The mediation of competitive interactions in *Antennaria dioica*^38^ by arbuscular mycorrhiza has been thoroughly studied; females have been found to have higher levels of AM colonization and benefit more from AM fungi than males in terms of growth and reproduction. The extent to which microbial factors drive sex-specific effects on the growth of *D. morrisiana* seedlings remains unknown and warrants further studies in the field.

## Methods

### Plant materials and growth

*Diospyros morrisiana* Hanse (Ebenaceae) is a dioecious subtropical tree growing in eastern Asia, particularly in southern China and Japan^39,40^. Seeds were collected from two *D. morrisiana* trees: tree A in Heishiding Natural Reserve (23.27°N, 111.15°E, Guangdong Province, China) and tree B in Jianfengling Natural Reserve (18°N, 108°E, Hainan Province, China). The seeds were marked as seeds A or B, corresponding to the parent tree.

The seeds were soaked in sterile water for 30 min and surface-sterilized with 15% hydrogen peroxide for 20 min. The sterilized seeds were pregerminated in petri dishes with 0.1 L growth medium for 5 days at 30 °C in the dark. After germination, the seedlings were transplanted to a transparent 3D growth cylinder 20 cm in height and 10 cm in diameter, which was filled with 1.2 L growth medium consisting of half-strength Hoagland solution solidified by 0.2% Phytagel^TM^ (Sigma-Aldrich, Germany)^41^. The distance between two neighboring plants was 2 cm^7^. Cylinders were placed in growth incubators at 26 °C during the day and 21 °C at night in a 12 h:12 h (day:night) cycle for 7 weeks, while the lower half of the cylinders was covered by aluminum foil to simulate a dark underground environment.

### Cultivation design

Seedlings from the same mother tree were regarded as kin (Combinations: A-A and B-B), while seedlings from different mother trees were regarded as nonkin (Combinations: A-B). The sexual interactions included intersexual (female–male, F-M) and intrasexual (F-F and M-M) interactions. In the single plantings, only one seedling was planted in each container as the control group. There were 22 replicates for the single planting treatment (13 females, 9 males), 54 replicates for the kin pair treatments, and 12 replicates for the nonkin pair treatments (total *N* = 154). Sexual identity could only be identified after harvest by a sex-specific molecular marker method (following section). The valid independent sexual combinations identified postharvest were 29 F-F, 27 F-M, and 10 M-M.

### Sex identification

The sex of dioecious plants is difficult to determine during early developmental stages if the plants are not in bloom or bearing fruit. For *Diospyros* plant species, OGI DNA markers, which control the expression of maleness^42^, are regarded as an effective method to identify sex in the species. OGI encodes a small RNA that in turn triggers transitive RNAi on a feminizing gene. This OGI marker was used to identify the sexuality of *D. morrisiana* seedlings and to further test the accuracy of OGI DNA markers, we observed 271 *D. morrisiana* mature trees and identified their sex according to flowering or fruiting in Heishiding Natural Reserve in 2017. We collected the leaves of these trees for DNA extraction.

The OGI marker primers were OGI-candF1 (5′-CACAGTAGTCATATATTTTTAGC-3′) and OGI-spR (5′-CTGGCA CACAAAATATTTTCAACCCT-3′)^43^. The PCR mixture contained a total volume of 20 µl, including 120 ng template DNA, 600 nM forward and reverse primers and 10 µl 2 × EasyTaq PCR Super Mix(+dye) (Transgene, Guangzhou, China). The OGI amplification steps consisted of an initial denaturing step at 94 °C for 3 min, followed by 35 cycles at 94 °C for 30 s, 58 °C for 30 s, 72 °C for 90 s, and a final step at 72 °C for 7 min. The results showed that the OGI markers indicate the sex of *D. morrisiana* with a 98.6% accuracy.

### Chromatography-mass spectrometry (GC-MS) analysis of root exudates

For each sex, we collected 6 root exudate gel samples from the in situ culture systems of the 7-week-old seedlings under aseptic conditions: 10 ml of each sample was sealed in a sample bottle, a solid-phase microextraction fiber was applied at 30/50 u, followed by a water bath at 50–60 °C, a headspace extraction for 40 min, and 250 °C desorption for 3 min. The derivatives were analyzed by a Trace 2000 gas chromatograph containing a 30-m-long, 0.25-mm inner diameter rtx5Sil-MS column with an additional 10-m integrated guard column. The GC injection temperature was 50 °C, ramped to 250 °C at 4 °C/min, and finally held constant for 15 min^44^. The carrier gas was high-purity helium. The mass spectrometer was used with a unit mass resolution at 17 spectra s^−1^ from 80 to 500 Da at −70 eV ionization energy and 1800 V detector voltage with a 230 °C transfer line and a 250 °C ion source. Metabolites were automatically detected with the BinBase algorithm^45^ and unambiguously identified in comparison with the retention index and mass spectrum of each analyte against the Fiehn^45^ mass spectral library. All compounds appeared consistently across all samples and were therefore likely to be of biological origin. The peak area of each compound was normalized based on the internal standard and used for further comparisons.

### Plant growth trait measurements

Seedling growth was imaged in situ every 3 days after germination using a digital camera (Nikon D600, EFS 60 mm, Japan). The seedlings were harvested 7 weeks after germination. ImageJ software (version 1.49, National Institutes of Health, USA) and WinRHIZO (Pro 2013a, Regent Instrument Inc., Canada) were used to measure a range of morphological traits (Table S1). The shoot and root dry biomass (mg) were measured after drying in an oven at 75 °C for 48 h. The specific root length was calculated as the ratio of the root length to the root dry biomass. The root/shoot biomass ratio was calculated by dividing the root dry biomass by the shoot dry biomass.

### Root spatial distribution measurements

We defined roots present in the resource zone as overlapping and calculated the ratio of the overlapping root length to the total root length (ROR) (Eq. (1)) to evaluate the spatial distribution of roots. *L*_*a*_ is the maximum root depth, *L*_*b*_ is the maximum root width of two plants, and *L*_*b*_′ is the seed distance between two plants (Fig. S1):

1 $${\mathrm{ROR}} = L_{12}/\left( {L_1 + L_2} \right)$$

where *L*_12_ is the total root length in the overlapping resource zone (region *L*_*a*_ *×* *L*_*b*_′) and *L*_1_ and *L*_2_ represent the total root length of each plant in the *L*_*a*_ *×* *L*_*b*_ region, respectively. ROR has values ranging from 0 to 1. When ROR is 0, the root systems are mutually exclusive, and no roots are distributed in the resource-overlapping zone; when ROR is 1, the root systems are completely intertwined.

### Statistical analyses

First, we analyzed our experiment by using ANOVA to test the effects of sex interactions (F-M, F-F, and M-M), kinship (kin and nonkin), and their interactions on the growth traits and ROR of *D. morrisiana*. Due to the unbalanced study design, post hoc multiple comparisons of trait parameters among different relatedness and sexual interaction treatments were used, and least-squares means were calculated using the R package “lsmeans”^46^.

Second, to compare the difference in root growth dynamics among planting treatments, we modeled the root width and depth of *D. morrisiana* as a function of plant age using the three-parameter logistic model proposed by Paine et al.^47^ for each treatment. We also obtained the function-derived absolute growth rate (AGR) and relative growth rate (RGR) from each dynamic model to further visualize the growth dynamics of roots. All statistical analyses were performed using R, Version 3.4.2^48^.

Finally, the results of the peak area of compounds were expressed as the mean value ± standard deviation and were compared using an analysis of variance (one-way ANOVA) for multiple comparisons.

## Supplementary information

Supporting information

## Data Availability

The data supporting the findings in this study are available from the corresponding author upon reasonable request. The source data are provided with this paper.
