# Supplementary material for: Sexual competition and kin recognition co-shape the traits of neighboring dioecious Diospyros morrisiana seedlings
Source: Hortic Res. 2021 Jul 1;8:162. doi: 10.1038/s41438-021-00598-9 (PMC8245536; doi:10.1038/s41438-021-00598-9)
Supplement: Supplementary file 1 — Supporting information [file 41438_2021_598_MOESM1_ESM.docx]

**Supporting information**

**Table S1** The average value ± error of female and male seedlings traits under monoculture and pair culture. *, *p* < 0.05; **, *p* < 0.01; ***, *p* <0.001.

| **Traits** | **Monoculture** | | |  | **Pair culture** | | |
| --- | --- | --- | --- | --- | --- | --- | --- |
|  | **Female** | **Male** | ***P*-value** |  | **Female** | **Male** | ***P*-value** |
| Shoot biomass (mg) | 27.24±1.83 | 32.83±2.69 | 0.1090 |  | 32.79±1.13 | 33.35±1.82 | 0.7830 |
| Root biomass (mg) | 10.52±0.71 | 14.66±1.88 | **0.0185*** |  | 12.14±0.35 | 11.85±0.46 | 0.6040 |
| Root/Shoot biomass | 0.39±0.02 | 0.46±0.07 | 0.2390 |  | 0.38±0.01 | 0.38±0.02 | 0.7130 |
| Leaf biomass (mg) | 17.48±1.43 | 19.88±1.47 | 0.3360 |  | 20.92±0.94 | 19.49±0.84 | 0.2790 |
| Stem biomass (mg) | 9.76±0.69 | 12.96±1.68 | **0.0457*** |  | 11.86±0.47 | 13.86±1.67 | 0.1940 |
| Total biomass (mg) | 37.77±2.37 | 47.49±3.38 | **0.0338*** |  | 44.93±1.37 | 45.20±1.95 | 0.9080 |
| Shoot height (cm) | 4.79±0.24 | 5.77±0.27 | **0.0253*** |  | 5.04±0.13 | 4.92±0.13 | 0.5270 |
| Total root length (cm) | 18.33±1.48 | 32.82±5.23 | **0.0015**** |  | 23.64±1.03 | 19.62±0.98 | **0.0071**** |
| Lateral root length (cm) | 4.77±1.42 | 19.85±5.38 | **0.0011**** |  | 9.66±1.04 | 6.04±0.81 | **0.0112*** |
| Tap root length (cm) | 13.56±0.81 | 12.97±0.69 | 0.6630 |  | 13.98±0.34 | 13.57±0.44 | 0.4650 |
| Total root length/Shoot height | 3.82±0.23 | 5.57±0.70 | **0.0054**** |  | 4.74±0.18 | 4.08±0.20 | **0.0160*** |
| Lateral root number | 1.82±0.56 | 6.71±1.04 | **0.0002***** |  | 3.43±0.34 | 2.54±0.29 | 0.0557 |
| Per lateral root length | 1.07±0.29 | 3.07±0.69 | **0.00427**** |  | 2.60±0.24 | 2.06±0.23 | 0.1220 |
| Root surface area (cm2) | 6.20±0.37 | 8.93±1.24 | **0.0098**** |  | 7.14±0.22 | 6.68±0.32 | 0.2160 |
| Root average diameter (mm) | 1.13±0.07 | 0.90±0.07 | 0.0610 |  | 1.02±0.03 | 1.11±0.03 | 0.0655 |
| Root volume(cm3) | 0.18±0.02 | 0.20±0.03 | 0.4820 |  | 0.18±0.01 | 0.19±0.01 | 0.8170 |
| Root length density (cm/cm3) | 18.33±1.48 | 32.82±5.23 | **0.0015**** |  | 23.64±1.03 | 19.62±0.98 | **0.0071**** |
| Root tissue density(mg/cm3) | 63.26±3.65 | 81.99±16.41 | 0.1240 |  | 71.44±2.19 | 69.49±2.74 | 0.5740 |
| Specific root length (cm/g) | 1.83±0.17 | 2.35±0.36 | 0.1550 |  | 2.01±0.09 | 1.68±0.06 | **0.0077**** |

# Table S2 Summary of the effect of Gender (female, male), Kinship (siblings, strangers) and their interactions on plant morphological traits in the 7^th^ week *D. morrisiana* seedlings according to two-way ANOVA. Significant effect (*p*<0.05) are shown in bold.

| **Traits** | **Sex** | | |  | **Kinship** | | |  | **Sex * Kinship** | | |
| --- | --- | --- | --- | --- | --- | --- | --- | --- | --- | --- | --- |
|  | df | F value | Pr(>F) |  | df | F value | Pr(>F) |  | df | F value | Pr(>F) |
| Leaf biomass (mg) | 1 | 1.355 | 0.2465 |  | 1 | 2.338 | 0.1287 |  | 1 | 5.231 | 0.0238* |
| Root biomass (mg) | 1 | 0.05 | 0.824 |  | 1 | 1.797 | 0.182 |  | 1 | 0.000 | 0.984 |
| Total biomass (mg) | 1 | 0.017 | 0.8961 |  | 1 | 0.149 | 0.7001 |  | 1 | 2.785 | 0.0976 |
| Biomass allocation (Shoot/Root) | 1 | 0.816 | 0.3681 |  | 1 | 0.995 | 0.3203 |  | 1 | 5.501 | 0.0205* |
| Tap root length (cm) | 1 | 0.281 | 0.5969 |  | 1 | 4.57 | 0.0344* |  | 1 | 0.214 | 0.6446 |
| Lateral root length (cm) | 1 | 5.043 | 0.0264* |  | 1 | 0.108 | 0.7426 |  | 1 | 0.818 | 0.3675 |
| Total root length (cm) | 1 | 3.301 | 0.0716 |  | 1 | 1.394 | 0.2399 |  | 1 | 1.021 | 0.3141 |
| Lateral root number | 1 | 4.005 | 0.0475* |  | 1 | 0.05 | 0.8235 |  | 1 | 0.797 | 0.3736 |
| root length density (cm/m^3^) | 1 | 5.336 | 0.0225* |  | 1 | 0.014 | 0.9059 |  | 1 | 0.026 | 0.8710 |
| SRL (cm/mg) | 1 | 2.966 | 0.0874 |  | 1 | 0.601 | 0.4395 |  | 1 | 0.097 | 0.7558 |


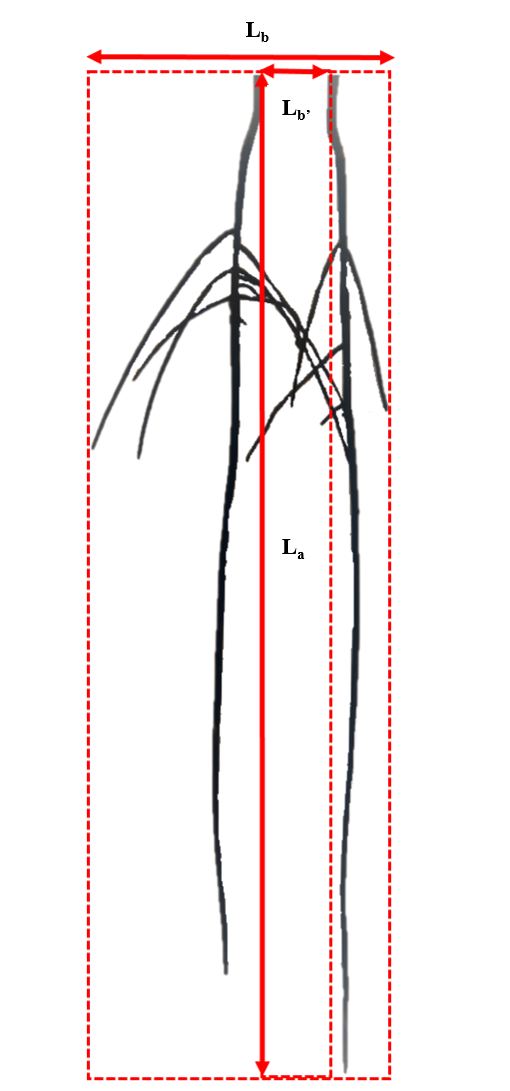


**Figure S1** Calculation of the ROR index. ROR quantifies the ratio overlapped root length in the resource utilization overlap zone to total root length. is the maximum root depth, is the maximum root width of two plants, is the distance between the seeds of the two plant individuals.
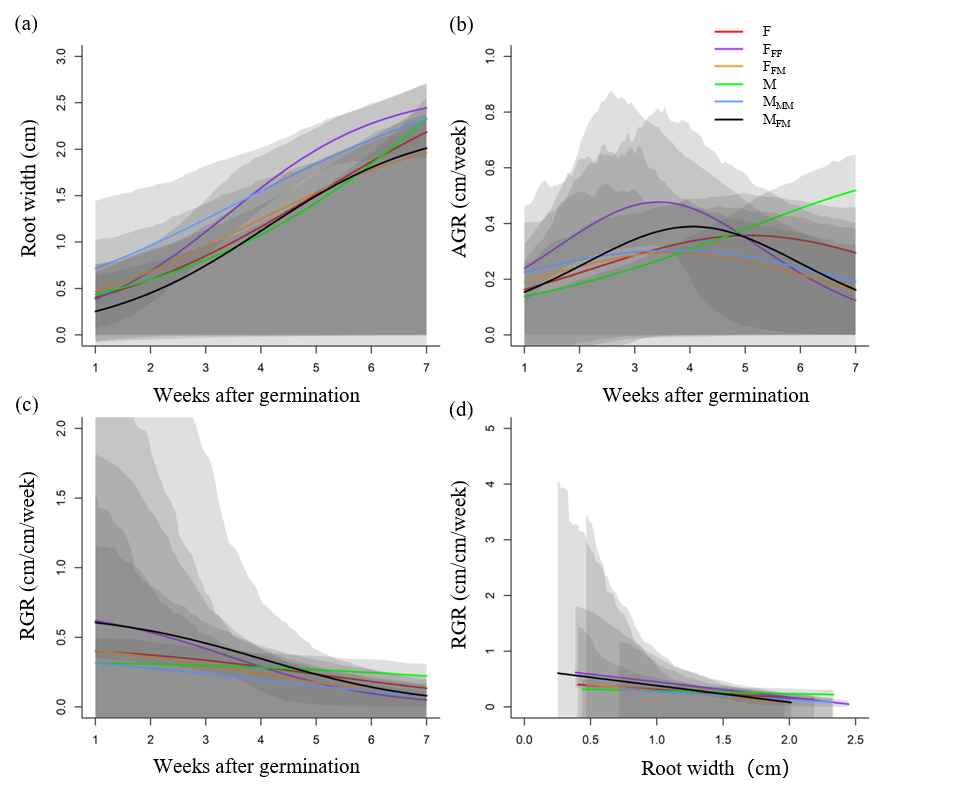


Female

**Figure S2** Predicted values of *D. morrisiana* Predicted root width growth of *D. morrisiana* from logistic model when grown in monoculture and in combination with different genders. (a) root width, (b) absolute root width growth rate (AGR), (c) relative root width growth rate (RGR) on the time basis, (d) relative growth rate (RGR) on the size basis. F represents single female, F_FF_ represents female in the intra-sexual interactions, F_FM_ represents female in the inter-sexual interactions, M represents single male, M_MM_ represents male in the intra-sexual interactions, M_FM_ represents male in the inter-sexual interactions. Grey curve indicates 95% confidence intervals for the accumulate growth and the growth rates, as derived from population prediction intervals.

# (a) Biomass


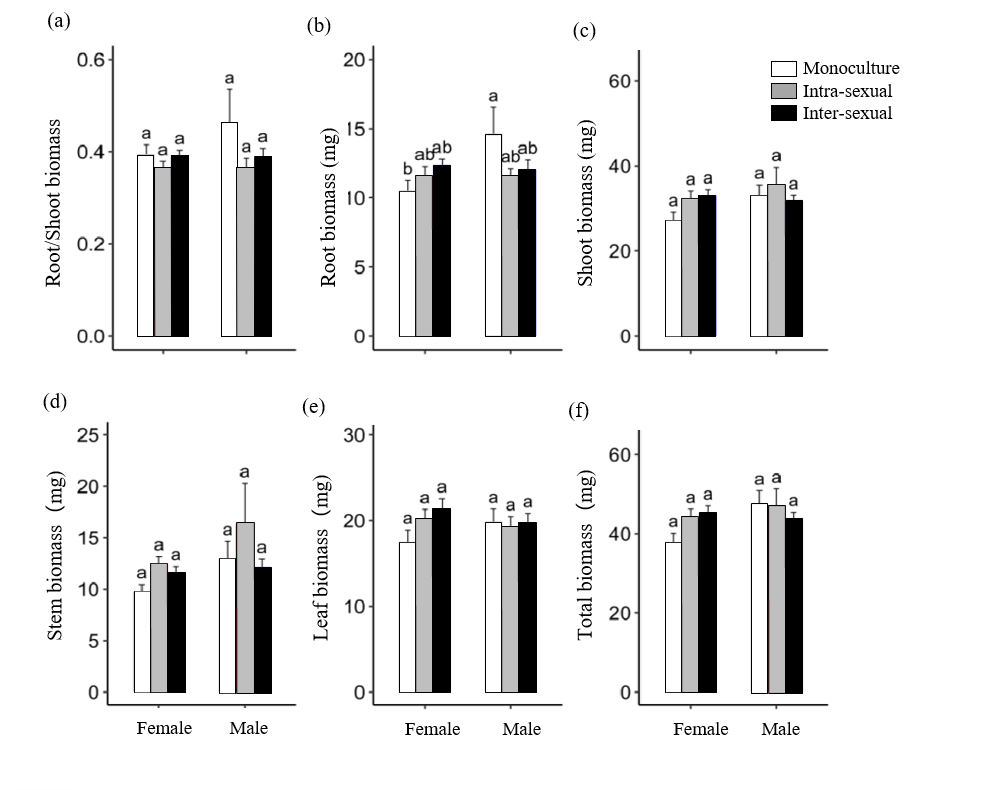


(b) Traits


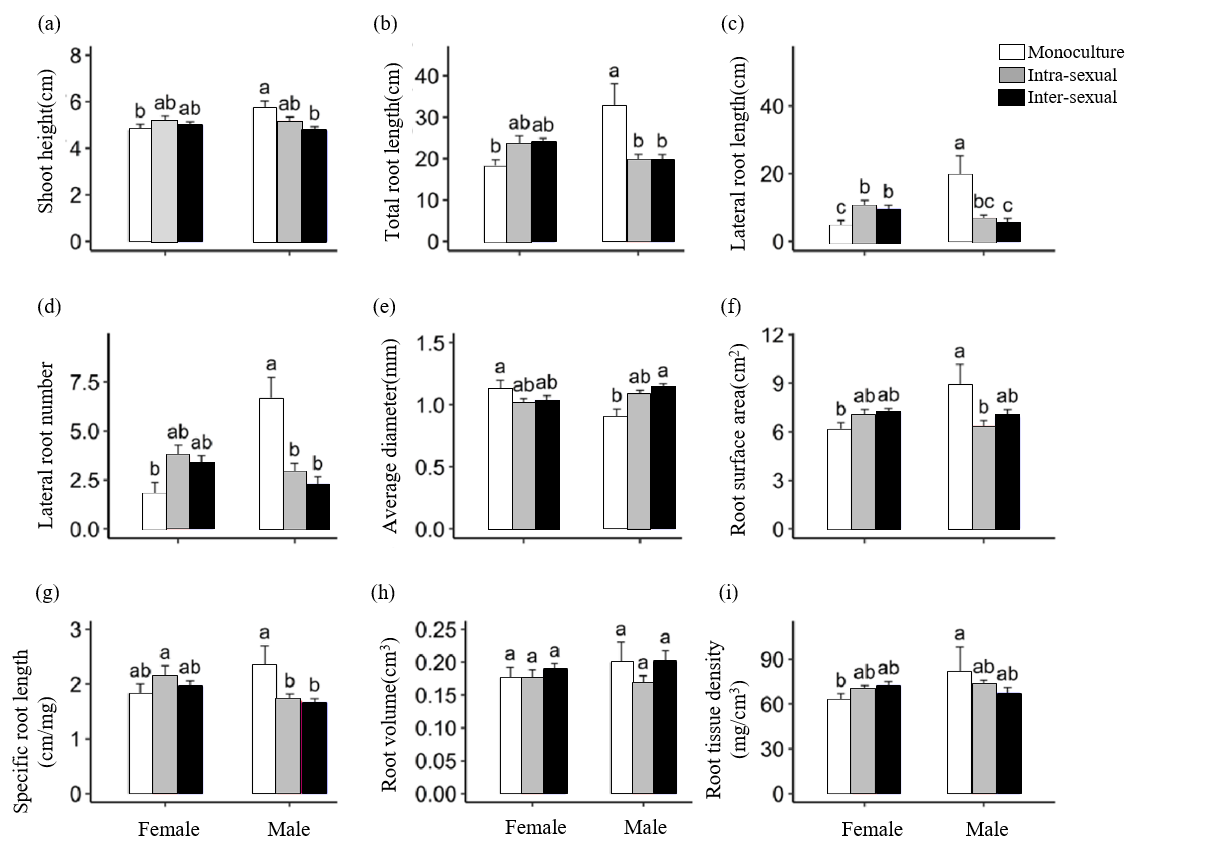


**Figure S3** Effects of competition for each gender on (a) biomass and (b) traits. Bar height indicates the mean value over all replicates, with error bars showing +1 SE are given without data transformation. Different letters indicate statistically significant differences (*p* < 0.05) among sex competition treatments.

**Table S3** Compounds in root exudates of 7-week *Diospyros morrisiana* seedlings

*Area Pct refers to the percentage of one or some peak areas in the total peak area in the chromatogram.*

| **Compounds (detected in more than two samples of each gender)** | | **Molecular formula** | **Area Pct** | |
| --- | --- | --- | --- | --- |
|  |  |  | **Female** | **Male** |
| **Ⅰ. only detected in female root exudates** | | | | |
| 1 | 3-Hydroxymandelic acid, ethyl ester, di-TMS | C_10_H_12_O_3_ | 2.89 |  |
| 2 | 1,2-Benzenedicarboxylic acid, bis(2-methylpropyl) ester | C_16_H_22_O_4_ | 2.09 |  |
| 3 | Benzaldehyde, 3,5-dimethyl- | C_9_H_10_O | 0.84 |  |
| 4 | Hexasiloxane, tetradecamethyl- | C_14_H_42_O_5_Si_6_ | 0.56 |  |
| 5 | Octasiloxane,1,1,3,3,5,5,7,7,9,9,11,11,13,13,15,15-hexadecamethyl- | C_16_H_50_O_7_Si_8_ | 0.56 |  |
| 6 | Pyridine | C_5_H_5_N | 0.44 |  |
| 7 | Phenol,3-(1,1-dimethylethyl)-4-methoxy- | C_11_H_16_O_2_ | 0.30 |  |
| 8 | 2-Hexene,2,5,5-trimethyl- | C_9_H_18_ | 0.14 |  |
| **Ⅱ. Only detected in male root exudates** | | | | |
| 9 | Undecanal | C_11_H_22_O |  | 0.44 |
| 10 | Octadecanal | C_18_H_36_O |  | 0.44 |
| 11 | Oxirane, tetradecyl- | C_17_H_34_O_2_ |  | 0.41 |
| 12 | Hexanoic acid, 3,5,5-trimethyl-, pentyl ester | C_14_H_28_O_2_ |  | 0.38 |
| **Ⅲ. both detected in female and male root exudates** | | | | |
| 13 | Silanediol, dimethyl- | C_2_H_8_O_2_Si | 26.39 | 21.81 |
| 14 | Hexadecane,2,6,10,14-tetramethyl- | C_20_H_42_ | 13.82 | 8.95 |
| 15 | 2,4-Di-tert-butylphenol | C_14_H_22_O | 13.75 | 7.26 |
| 16 | Phthalic acid, isobutyl trans-hex-3-enyl ester | C_16_H_22_O_4_ | 8.75 | 8.81 |
| 17 | Oxime-，methoxy-phenyl- | C_8_H_9_NO_2_ | 7.73 | 5.97 |
| 18 | Cyclotetrasiloxane，octamethyl- | C_8_H_24_O_4_Si_4_ | 6.76 | 7.91 |
| 19 | Cyclotrisiloxane，hexamethyl- | C_6_H_18_O_3_Si_3_ | 6.26 | 7.29 |
| **Ⅲ. both detected in female and male root exudates** | | | | |
| 20 | 1,3,5,7-Tetraethyl-1-ethylbutoxysiloxycyclotetrasiloxane | C_14_H_34_O_6_Si_5_ | 3.98 | 1.25 |
| 21 | Di-sec-butyl phthalate | C_16_H_22_O_4_ | 3.63 | 2.48 |
| 22 | 2,5-Dihydroxybenzoic acid, 3TMS derivative | C_7_H_6_O_4_ | 3.40 | 3.67 |
| 23 | Pentadecane, 2,6,10,14-tetramethyl- | C_19_H_40_ | 2.61 | 0.10 |
| 24 | Benzaldehyde,2,4-dimethyl- | C_9_H_10_O | 2.43 | 5.07 |
| 25 | Cyclopentasiloxane, decamethyl- | C_10_H_30_O_5_Si_5_ | 2.34 | 2.53 |
| 26 | Acetic acid | C_2_H_4_O_2_ | 2.22 | 1.81 |
| 27 | Nonanal | C_9_H_18_O | 2.05 | 8.56 |
| 28 | Decanal | C_10_H_20_O | 1.91 | 6.97 |
| 29 | Benzaldehyde,3,4-dimethyl- | C_9_H_10_O | 1.77 | 2.43 |
| 30 | Octadecane | C_18_H_38_ | 1.23 | 0.80 |
| 31 | 3,4-Dihydroxyphenylglycol, 4TMS derivative | C_8_H_10_O_4_ | 1.11 | 6.98 |
| 32 | Cyclohexasiloxane, dodecamethyl- | C_12_H_36_O_6_Si_6_ | 1.09 | 1.25 |
| 33 | Pentasiloxane, dodecamethyl- | C_12_H_36_O_4_Si_5_ | 0.98 | 0.69 |
| 34 | 1,1,1,5,7,7,7-Heptamethyl-3,3-bis(trimethylsiloxy)tetrasiloxane | C_12_H_36_O_5_Si_6_ | 0.96 | 3.23 |
| 35 | 2,5-cyclohexadien-1-one, 2,6-bis(1,1-dimethylethyl)-4-hydroxy-4-methyl- | C_15_H_24_O_2_ | 0.88 | 0.85 |
| 36 | 5,9-Undecadien-2-one,6,10-dimethyl-, (E)- | C_13_H_22_O | 0.79 | 0.93 |
| 37 | 5,9-Undecadien-2-one,6,10-dimethyl-, (E)- | C_13_H_22_O | 0.79 | 0.93 |
| 38 | Nonadecane, 9-methyl- | C_20_H_42_ | 0.65 | 0.23 |
| 39 | Dibutyl phthalate | C_16_H_22_O_4_ | 0.63 | 2.09 |
| 40 | Heptadecane | C_17_H_36_ | 0.56 | 0.39 |
| 41 | N-Benzyl-N-ethyl-p-isopropylbenzamide | C_19_H_23_NO | 0.47 | 0.39 |
| 42 | Benzoic acid, 2-ethylhexyl ester | C_15_H_22_O_2_ | 0.35 | 0.53 |
| 43 | Nonadecane | C_19_H_40_ | 0.33 | 0.46 |
| **Ⅲ. both detected in female and male root exudates** | | | | |
| 44 | Benzaldehyde, 2,4,5-trimethyl- | C_10_H_12_O | 0.27 | 0.49 |
| 45 | Trisiloxane,1,1,1,5,5,5-hexamethyl-3,3-bis[(trimethylsilyl)oxy]- | C_12_H_36_O_4_Si_5_ | 0.24 | 0.67 |
| 46 | 2,2,4-Trimethyl-1,3-pentanediol diisobutyrate | C_16_H_30_O_4_ | 0.14 | 0.98 |


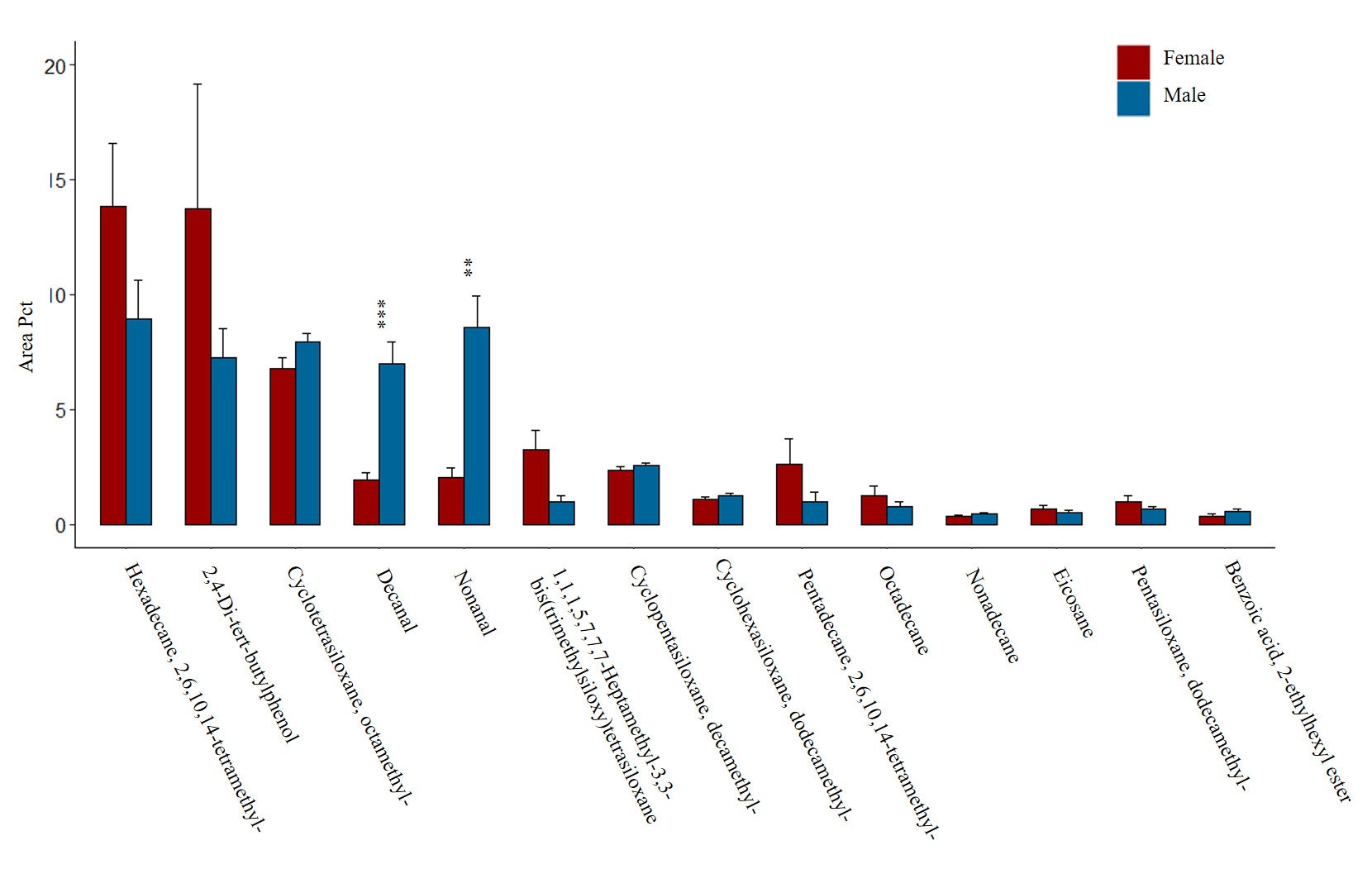


**Figure S4** Peak area percentages of root exudates compounds which detected in more than 3 samples of each gender. Mean+1 SE are given without data transformation. Asterisk indicate statistically significant differences (* *p* < 0. 01, ** *p* < 0.05, *** *p* < 0.001) between female and male.
